# Bacterial distributions and prognosis of bloodstream infections in patients with liver cirrhosis

**DOI:** 10.1038/s41598-017-11587-1

**Published:** 2017-09-13

**Authors:** Yangxin Xie, Bo Tu, Zhe Xu, Xin Zhang, Jingfeng Bi, Min Zhao, Weiwei Chen, Lei Shi, Peng Zhao, Chunmei Bao, Enqiang Qin, Dongping Xu

**Affiliations:** 1 Medical School of Chinese PLA, Beijing, China; 2 Treatment and Research Center for Infectious Diseases, Beijing 302 Hospital, Beijing, China; 3 Research Center for Clinical & Translational Medicine, Beijing 302 Hospital, Beijing, China; 4 Clinical Laboratory Center, Beijing 302 Hospital, Beijing, China

## Abstract

Bloodstream infections (BSIs) are a frequently observed complication in liver cirrhosis patients. This study aimed to investigate the microbiological characteristics and outcomes of BSIs in patients with liver cirrhosis. We retrospectively studied 852 patients with liver cirrhosis who developed a BSI. Patient outcome was evaluated using 30-day mortality and assessed using multivariate stepwise logistic regression analysis. Antibiotic sensitivity of the pathogens was tested. Gram-negative bacteria were responsible for 59.6% of BSIs, and Gram-positive bacteria caused 40.4% of the episodes among liver cirrhosis patients. The bacterial distribution significantly differed between hospital-acquired and community-acquired infections, especially in cases caused by Gram-negative pathogens. The results of the drug sensitivity test suggested that amikacin, cefoperazone/sulbactam, and piperacillin/tazobactam highly suppressed Gram-negative infections, while vancomycin and teicoplanin strongly inhibited Gram-positive BSIs. Liver failure, liver cancer, complications, Child-Pugh grade, septic shock, administration of appropriate antibiotics within 24 h, ICU admission, nosocomial infection, and Gram nature of the bacteria were independent risk factors for 30-day mortality (*P* < 0.05). The choice of initial empirical antibiotics should be based on the type, severity and origin of infection and on the local epidemiological data on antibiotic resistance. Accurate evaluation of risk factors for mortality may improve appropriate therapeutic choice.

## Introduction

Liver cirrhosis is one of the leading causes of death worldwide^1^. Hepatitis B virus (HBV), hepatitis C virus, alcoholism, and non-alcoholic fatty liver disease are the most common conditions leading to liver cirrhosis^2^. China has the highest HBV infection burden in the world. According to statistics, there are approximately 120 million hepatitis B surface antigen (HBsAg) carriers, and nearly 300,000 individuals die from HBV-related liver diseases each year in China^3^. Patients with liver cirrhosis have been found to be more likely to acquire bacterial infections due to their dysregulated immune function^4^. Bloodstream infections (BSIs) are frequently observed complications in liver cirrhosis patients^5^. BSIs in cirrhosis patients are associated with prolonged hospital stay, rapid progression of liver disease, poor prognosis, and an increased risk of mortality^6^. Timely and appropriate empirical antibiotic therapy is pivotal for BSI management^7^. However, the heterogeneous epidemiology of BSI may increase the difficulty of empirical antibiotic management, especially with the increasing prevalence of Gram-positive and multidrug-resistant bacteria^8, 9^.

A growing body of evidence indicates that the source of infection may influence the bacterial distribution and sensitivity of these bacteria to antibiotics in liver cirrhosis patients who develop BSIs^10, 11^. Several studies have found that liver cirrhosis patients who have nosocomial infections exhibit high resistance to empirical antibiotic treatments^12–14^. Therefore, the choice of antimicrobial coverage should be take into account local epidemiology and the site of infection onset. Improved knowledge of the local epidemiology of bacterial infections is necessary. However, few such retrospective studies with a large sample size have been reported in China.

In this study, we aimed to investigate the distribution of both Gram-negative and Gram-positive bacteria and their sensitivity to commonly used antibiotics in liver cirrhosis patients who develop BSIs. In addition, we defined the risk factors for 30-day mortality in the study population.

## Results

### Basic characteristics of the study population

Patients with liver cirrhosis who developed BSIs during the study period were recruited to participate in this study. In total, 852 liver cirrhosis patients participated in the present study, including both men (78.8%) and women (21.2%), with an average age of 50.80 ± 11.78 years. Of these, 155 (18.2%) were admitted to the ICU (Table 1).

**Table 1:** Basic characteristics of the study population.

Features	Total (n = 852)	Survivors, n = 660 (77.5%)	Non-survivors 192 (22.5%)	*P* value
**Demographic characteristics**				
Male (n, %)	671 (78.8)	528 (80)	143 (74)	0.04
Age (years)	50.8 ± 11.8	50.6 ± 11.4	51.5 ± 13.2	0.366
Hospitalization unit				<0.001
General ward (n, %)	697 (81.8)	595 (90.2)	102 (53.1)	
ICU (n, %)	155 (18.2)	65 (9.9)	90 (46.9)	
**Liver diseases**				0.631
Single Hepatitis B (n, %)	548 (64.3)	429 (65.0)	119 (62.0)	
Single Hepatitis C (n, %)	85 (10.0)	68 (10.3)	17 (8.9)	
Combined with Hepatitis B and C (n, %)	8 (0.9)	7 (1.1)	1 (0.5)	
Alcoholic (n, %)	107 (12.6)	80 (12.1)	27 (14.1)	
Others (n, %)	104 (12.2)	76 (11.5)	28 (14.6)	
Combined with liver failure (n, %)	188 (22.1)	95 (14.4)	93 (48.4)	<0.001
Combined with hepatocellular carcinoma (n, %)	250 (29.3)	202 (30.6)	48 (25.0)	0.133
**Child-Pugh classification**				<0.001
Class A (n, %)	109 (12.8)	105(15.9)	4 (2.1)	
Class B (n, %)	302 (35.4)	267 (40.5)	35 (18.2)	
Class C (n, %)	441 (51.8)	289 (43.8)	152 (79.2)	
**BSI data**				
Nosocomial infection (n, %)	523 (61.4)	390 (59.1)	134 (69.8)	0.007
**Infection history within 2 years**	455 (53.4)	347 (52.6)	108 (56.3)	0.369
**BSI source**				<0.001
Primary (n, %)	513 (60.2)	427 (64.7)	86 (44.8)	
Lung (n, %)	48 (5.6)	21 (3.2)	27 (14.1)	
Abdominal (SBP) (n, %)	277 (32.5)	199 (30.2)	78 (40.6)	
Urinary tract (n, %)	7 (0.8)	7 (1.1)	0 (0.00)	
**Complications**				<0.001
Ascites (n, %)	486 (57.0)	325 (49.2)	161 (83.9)	
Upper gastrointestinal bleeding (n, %)	58 (6.8)	28 (4.2)	30 (15.6)	
Hepatic encephalopathy (n, %)	118 (13.9)	72 (10.9)	46 (24.0)	
Hepato-renal syndrome (n, %)	27 (3.2)	7 (1.1)	20 (10.4)	
More than one complication (n, %)	172 (20.2)	46 (7.0)	126 (65.6)	
Septic shock	161 (18.9)	58 (8.8)	103 (53.7)	<0.001
**Appropriate antibiotics within 24 h** (n, %)	612 (72.8)	501 (75.9)	111 (57.8)	<0.001

Note: ICU: Intensive care unit; BSI: Bloodstream infection.

We evaluated the types of liver cirrhosis based on the study population. Hepatitis B was the most common cause of liver disease in the study population (64.3%), followed by alcoholic liver disease and hepatitis C. Furthermore, 22.1% of the patients with liver cirrhosis presented with liver failure, while 29.3% had hepatocellular carcinoma. Few patients were classified as Child-Pugh class A (12.8%), with the majority of patients classified as class B (35.4%) or class C (51.8%) chronic liver disease (Table 1).

We summarized the medical information of BSI in liver cirrhosis patients. In our study, 61.4% of patients were diagnosed with nosocomial infection. Approximately half of the patients (53.4%) had a history of infection within two years. Primary infection was the major reason for BSI, accounting for 60.2% of the cases, followed by spontaneous bacterial peritonitis (32.5%), lung infection (5.6%), and urinary tract infection (0.8%). The most frequent complications of BSI in liver cirrhosis were ascites, hepatic encephalopathy, upper gastrointestinal bleeding, and hepatorenal syndrome. In addition, 172 patients presented with more than one type of complication. The occurrence rate of septic shock was 18.9% (Table 1).

Additionally, empirical therapy was considered adequate when at least one active antibiotic against the isolated pathogen, according to the species identification and susceptibility test, was administered during the first 24 h after blood cultures were drawn (before microbiological results were available). Overall, 72.8% of the patients received adequate empirical antibiotics within 24 h (Table 1).

### Comparison of clinical characteristics between survivors and non-survivors

The 30-day mortality was used to estimate the primary clinical outcomes of liver cirrhosis patients developing BSIs. Among the study subjects, the mortality rate was 22.54%. We also analysed the clinical characteristics of the patients according to their survival status within 30 days after BSI. The results demonstrated that gender and ICU admission combined with liver failure, Child-Pugh score, nosocomial infection, BSI source, complications, and septic shock significantly differed between survivors and non-survivors (*P* < 0.05). Furthermore, markedly higher percentages of patients received adequate antibiotics within 24 h after infection in the survival group (*P* = 0.000; Table 1). Additionally, types of liver disease and occurrence of hepatocellular carcinoma did not significantly differ between the survivor and non-survivor groups (*P* > 0.05; Table 1).

### Bacterial distribution

In total, 852 cultures were isolated from the blood specimens. Of these, 59.6% were confirmed to be Gram-negative, while 40.4% were Gram-positive. The Gram-negative bacteria mainly included *Escherichia coli*, *Klebsiella pneumoniae*, *Aeromomas species*, *Enterobacter cloacae*, *Acinetobacter baumanii*, *Pseudomonas aeruginosa*, *Stenotrophomonas maltophilia*, and others. Furthermore, 138 isolated Gram-negative bacteria were identified as extended-spectrum beta-lactamase (ESBL)-positive, and 215 cultures were confirmed as ESBL-negative. Overall, 40.2% of *Escherichia coli* and *Klebsiella pneumoniae* presented as ESBL-positive, and the rest (59.8%) were negative. Gram-positive organisms included coagulase-negative staphylococci, *Streptococcus* spp., *Staphylococcus aureus*, and *Enterococcus spp*. Table 2 presents a detailed distribution of the bacteria.

**Table 2:** Comparison of bacterial distributions between survivors and non-survivors based on the study population.

Bacterial distributions	Total, n = 852	Survivors, n = 660 (77.5%)	Non-survivors n = 192 (22.5%)	*P* value
**Gram-negative bacteria**	508 (59.6)	377 (57.1)	131 (68.2)	0.006
*Escherichia coli*	245 (28.8)	185 (28.0)	60 (31.3)	0.519
*Klebsiella pneumoniae*	98 (11.5)	72 (10.9)	26 (13.5)	0.851
*Pseudomonas aeruginosa*	9 (1.1)	5 (0.8)	4 (2.1)	0.197
*Enterobacter cloacae*	19 (2.2)	14 (2.1)	5 (2.6)	0.957
*Aeromonas species*	30 (3.5)	24 (3.6)	6 (3.1)	0.455
*Acinetobacter baumanii*	16 (1.9)	6 (0.9)	10 (5.2)	0.001
*Stenotrophomonas maltophilia*	6 (0.7)	3 (0.5)	3 (1.6)	0.173
Others	75 (8.8)	61 (9.2)	14 (7.3)	0.127
Mixed	10 (1.2)	7 (1.1)	3 (1.6)	0.758
**ESBL status of the isolated pathogens**				0.061
ESBL (+)	138 (16.2)	97 (14.7)	41 (21.4)	
ESBL (−)	215 (25.2)	170 (25.8)	45 (23.4)	
**Gram-positive organisms**	344 (40.4)	283 (42.9)	61 (31.8)	0.006
***Coagulase-negative staphylococc*** *i*	158 (18.5)	131 (19.9)	27 (14.1)	0.398
*Staphylococcus epidermidis*	61 (7.2)	47 (7.1)	14 (7.3)	
*Staphylococcus huminis*	32 (3.8)	29 (4.4)	3 (1.6)	
*Staphylococcus haemolyticus*	19 (2.2)	16 (2.4)	3 (1.6)	
Others	46 (5.4)	39 (5.9)	7 (3.7)	
Methicillin resistance				0.235
Methicillin-resistant groups	74 (8.7)	60 (9.1)	14 (7.3)	
Methicillin-sensitive groups	61 (7.2)	54 (8.2)	7 (3.7)	
***Streptococcus species***	96 (11.3)	86 (13.0)	10 (5.2)	0.160
*Streptococcus pneumoniae*	12 (1.4)	9 (1.4)	3 (1.6)	
*Streptococcus gallolyticus*	23 (2.7)	22 (3.3)	1 (0.5)	
Others	61 (7.2)	55 (8.3)	6 (3.1)	
**Staphylococcus aureus**	58 (6.8)	46 (7.0)	12 (6.3)	—
Methicillin resistance				0.027
MRSA	8 (0.9)	4 (0.6)	4 (2.1)	
Methicillin-sensitive	45 (5.6)	38 (5.8)	7 (3.7)	
***Enterococcus spp***.	32 (3.8)	20 (3.0)	12 (6.3)	0.475
*Enterococcus faecium*	22 (2.6)	13 (2.0)	9 (4.7)	
*Enterococcus faecalis*	4 (0.5)	2 (0.3)	2 (1.0)	
Others	6 (0.7)	5 (0.8)	1 (0.5)	

Note: −: Indicates no related data; MRSA: methicillin-resistant Staphylococcus aureus; ESBL, extended-spectrum β-lactamase.

We analysed the methicillin resistance of coagulase-negative staphylococci and *S*. *aureus*. Overall, 74 coagulase-negative staphylococcal cultures and 8 *S*. *aureus* isolates were confirmed to be methicillin-resistant (Table 2). In Gram-positive bacteria, 23.8% were methicillin-resistant, and 9.3% were *Enterococcus spp*.

The results suggested that the distributions of Gram-positive and Gram-negative bacteria (*P* = 0.006), *Acinetobacter baumanii* (*P* = 0.001), and MRSA (*P* = 0.027) significantly differed between the survivor and non-survivor groups (Table 2).

### Effects of acquisition sites of infection on bacterial distribution

Epidemiological analyses were performed according to their acquisition sites of infection. As shown in Table 3, we found that the distributions of *Klebsiella pneumoniae* (*P* = 0.047), *Pseudomonas aeruginosa* (*P* = 0.011), *Enterobacter cloacae* (*P* = 0.022), *Stenotrophomonas maltophilia* (*P* = 0.039), and *Streptococcus* spp. (*P* = 0.035) significantly differed between nosocomial and community-acquired infections.

**Table 3:** Characteristics of isolated bacteria in liver cirrhosis patients suffering from nosocomial and community-acquired infections.

Bacterial distributions	Total, n = 852	Nosocomial infection, n = 523, 61.4%	Community-acquired infection, n = 329, 38.6%	*P* value
**Gram-negative bacteria**	508 (59.6)	299 (57.2)	209 (63.5)	0.058
*Escherichia coli*	245 (28.8)	135 (25.8)	110 (33.4)	0.097
*Klebsiella pneumoniae*	98 (11.5)	49 (9.4)	49 (14.5)	0.047
*Pseudomonas aeruginosa*	9 (1.1)	9 (1.7)	0 (0.00)	0.011
*Enterobacter cloacae*	19 (2.2)	16 (3.1)	3 (0.9)	0.022
*Aeromonas species*	30 (3.5)	15 (2.9)	15 (4.6)	0.309
*Acinetobacter baumanii*	16 (1.9)	14 (2.7)	2 (0.6)	0.018
*Stenotrophomonas maltophilia*	6 (0.7)	6 (1.2)	0 (0.00)	0.039
Others	75 (8.8)	52 (9.9)	23 (7.0)	0.046
Mixed	10 (1.2)	3 (0.6)	7 (2.1)	0.061
**ESBL status of the isolated pathogens**				0.302
ESBL (+)	138 (16.2)	79 (9.3)	59 (17.9)	
ESBL (−)	215 (25.2)	111 (13.0)	104 (31.6)	
**Gram-positive organisms**	344 (40.4)	224 (26.3)	119 (36.2)	0.058
***Coagulase-negative staphylococci***	158 (18.5)	111 (13.0)	47(14.3)	0.828
*Staphylococcus epidermidis*	61 (7.2)	43 (5.1)	18 (5.5)	
*Staphylococcus huminis*	32 (3.8)	23 (2.7)	9 (2.7)	
*Staphylococcus haemolyticus*	19 (2.2)	15 (1.8)	4 (1.2)	
Others	46 (5.3)	30 (3.5)	16 (4.9)	
Methicillin resistance				0.185
Methicillin-resistant groups	74 (8.7)	54 (6.3)	20 (6.1)	
Methicillin-sensitive groups	61 (7.2)	38 (4.5)	23 (7.0)	
***Streptococcus species***	96 (11.3)	43 (5.1)	43 (13.1)	0.035
*Streptococcus pneumoniae*	12(1.4)	5 (0.6)	7 (2.1)	
*Streptococcus gallolyticus*	23 (2.7)	18 (2.1)	5 (1.5)	
Others	61 (7.2)	30 (3.5)	31 (9.4)	
**Staphylococcus aureus**	58 (6.8)	38 (4.5)	20 (6.1)	—
**Methicillin resistance**				0.164
MRSA	8 (0.9)	7 (0.8)	1 (0.3)	
Methicillin-sensitive	45 (5.3)	28 (3.3)	17 (5.2)	
***Enterococcus spp***.	32(3.8)	20 (3.8)	12(3.6)	0.115
*Enterococcus faecium*	22 (2.6)	14(2.7)	8(2.4)	
*Enterococcus faecalis*	4 (0.5)	3 (0.6)	1(0.3)	
Others	6(0.7)	4 (0.8)	2(0.6)	

Note: −: Indicates no available data; MRSA: methicillin-resistant Staphylococcus aureus.

### Drug sensitivity analysis

We then investigated the sensitivity of the isolated cultures to commonly used antibiotics. The isolated Gram-negative bacterial strains were highly sensitive to amikacin, cefoperazone/sulbactam, meropenem, imipenem, and piperacillin/tazobactam, regardless of their ESBL status (Table 4).

**Table 4:** Drug resistance analysis for the isolated organisms.

Antibiotics	Isolated bacterium			
**Gram-negative bacteria**				
	All	Sensitivity n, %	ESBL + *Escherichia coli* (n/total, %)	ESBL + *Klebsiella pneumonia* (n/total, %)
Cefepime	462	351 (76.0)	27/121 (22.3)	4/8 (50.0)
Ceftazidime	480	352 (73.3)	21/122 (17.2)	1/8 (12.5)
Ceftriaxone	470	297 (63.2)	1/126 (0.8)	0/9 (0.0)
Levofloxacin	474	333 (70.3)	39/121 (32.2)	3/8 (37.5)
Cefoperazone/sulbactam	405	364 (89.9)	77/103 (74.8)	1/5 (20.0)
Piperacillin/tazobactam	467	428 (91.7)	100/118 (84.8)	6/9 (66.7)
Imipenem	485	464 (95.7)	126/126 (100)	9/9 (100.0)
Meropenem	387	373 (96.4)	104/105 (99.1)	8/9 (89.0)
Amikacin	473	457 (96.6)	119/125 (95.2)	8/9 (89.0)
**Gram-positive bacteria**				
	All	Sensitivity n, %	Methicillin-resistant *Coagulase-negative staphylococci*	MRSA
Penicillin	324	107 (33.0)	0/74 (0.0)	0/8 (0.0)
Ceftriaxone	182	113 (62.1)	0/47(0.0)	0/4 (0.0)
Levofloxacin	334	236 (70.7)	36/74 (48.7)	1/8 (1.3)
Erythromycin	316	89 (28.2)	11/74 (14.9)	4/8 (50.0)
Vancomycin	331	328 (99.1)	73/73 (100.0)	8/8 (100)
Teicoplanin	200	198 (99.0)	71/72 (98.6)	8/8 (100)

Drug sensitivity analysis of Gram-positive bacteria showed that these strains were highly sensitive to vancomycin and teicoplanin. In addition, methicillin-resistant coagulase-negative staphylococci and MRSA were also sensitive to vancomycin and teicoplanin (Table 4).

### Risk factors for 30-day mortality in liver cirrhosis patients combined with BSIs

In the current study, a stepwise logistics regression model was used to evaluate the prognostic significance of the clinical parameters for BSI in liver cirrhosis patients. The results of the univariate analyses demonstrated that gender, liver failure, non-primary infection source, presence of complications, Child-Pugh grade, septic shock, administration of appropriate antibiotics within 24 h, ICU admission, nosocomial infection, and Gram nature of the bacteria were significantly correlated with outcomes of liver cirrhosis patients who develop BSIs (Table 5).

**Table 5:** Univariate analysis of risk factors for 30-day mortality in liver cirrhosis patients suffering from BSIs.

Factor	Group	N	OR	90% CI		P value
Gender	M	671	1.000 (Reference)			
	F	181	1.479	1.018	2.149	0.0402
Age (years)	< = 44	251	1.000 (Reference)			
	45–59	519	1.014	0.700	1.467	0.9429
	> = 60	182	1.160	0.755	1.782	0.4930
Pathogen	Hepatitis B	548	1.000 (Reference)			
	Hepatitis C	85	0.840	0.483	1.460	0.5361
	Alcoholic	107	1.095	0.669	1.791	0.7193
	Other	104	1.374	0.866	2.181	0.1773
Combined with liver failure	No	664	1.000 (Reference)			
	Yes	188	5.949	4.164	8.499	<0.0001
Combined with liver cancer	No	602	1.000 (Reference)			
	Yes	250	0.749	0.519	1.080	0.1216
Infection history within 2 years	No	397	1.000 (Reference)			
	Yes	455	1.122	0.756	1.667	0.5679
Source of infection	Primary	513	1.000 (Reference)			
	Non-Primary	339	2.360	1.703	3.271	<0.0001
Complication	No	163	1.000 (Reference)			
	Yes	689	11.717	8.096	16.957	<0.0001
Child-Pugh grade	A	109	1.000 (Reference)			
	B	302	3.098	1.068	8.987	0.0375
	C	441	14.528	5.256	40.154	<0.0001
Septic shock	No	691	1.000 (Reference)			
	Yes	161	12.602	8.511	18.658	<0.0001
Appropriate antibiotics within24 h	Yes	612	1.000 (Reference)			
	No	240	6.060	4.282	8.576	<0.0001
ICU	No	697	1.000 (Reference)			
	Yes	155	9.092	9.092	13.396	<0.0001
Nosocomial infection	No	329	1.000 (Reference)			
	Yes	523	1.428	1.018	2.005	0.0392
Gram stain	Negative	508	1.000 (Reference)			
	Positive	344	0.633	0.451	0.888	0.0082

Note: Complication: including ascites or hepatorenal syndrome or hepatic encephalopathy or upper gastrointestinal bleeding. Combined with liver failure: cirrhosis with liver failure. Non-Primary: Sources of BSI including lung or urinary tract or abdomen.

Results of the multivariate stepwise logistic regression analyses identified liver failure, liver cancer, presence of complications, Child-Pugh grade, septic shock, administration of appropriate antibiotics within 24 h, ICU admission, nosocomial infection, and Gram nature of the bacteria as independent risk factors for 30-day mortality in the study population (Table 6).

**Table 6:** Multivariate analyses for 30-day mortality in the study population.

Factor	Group	N	OR	90% CI		P value
Combined with liver failure	No	664	1.000 (Reference)			
	Yes	188	2.288	1.451	3.609	0.0028
Combined with liver cancer	No	602	1.000 (Reference)			
	Yes	250	2.196	1.366	3.529	0.0064
Complication	No	163	1.000 (Reference)			
	Yes	689	4.663	3.057	7.113	<0.0001
Child-Pugh grade	A	109	1.000 (Reference)			
	B	302	5.483	1.914	15.708	0.0078
	C	441	10.499	3.687	29.894	0.0002
Septic shock	No	691	1.000 (Reference)			
	Yes	161	3.959	2.457	6.380	<0.0001
Appropriate antibiotics within 24 h	Yes	612	1.000 (Reference)			
	No	240	8.278	5.460	12.550	<0.0001
ICU	No	697	1.000 (Reference)			
	Yes	155	2.363	1.447	3.861	0.0039
Nosocomial infection	No	329	1.000 (Reference)			
	Yes	523	1.670	1.107	2.520	0.0402
Gram stain	Negative	508	1.000 (Reference)			
	Positive	344	0.465	0.305	0.710	0.0029

Note: Complication: including ascites or hepatorenal syndrome or hepatic encephalopathy or upper gastrointestinal bleeding. Combined with liver failure: cirrhosis with liver failure.

## Discussion

BSIs are a prevalent complication in liver cirrhosis patients and cause severe mortality. Compared to non-cirrhotic patients, cirrhotic patients have poor prognoses in BSIs^15, 16^. BSIs lead to poor patient outcomes^17, 18^, prolonged patient stays in the ICU and in the hospital, and substantial extra medical costs^19–21^. BSIs are associated with a higher mortality risk compared with pulmonary and intra-abdominal infections in patients with sepsis^22^. Timely and appropriate empirical antibiotic treatment is pivotal for the prognosis of liver cirrhosis patients suffering from BSIs. However, antibiotic management represents a great challenge in the clinical setting due to the heterogeneous aetiology of BSIs, the increasing prevalence of Gram-positive bacterial pathogens in BSIs, and the emergence of multidrug-resistant organisms^8, 9^. To improve the management of empirical antibiotic therapy, we investigated the clinical and epidemiological characteristics of BSIs in liver cirrhosis patients.

We found that Gram-negative bacteria were responsible for 59.6% of BSIs, and 40.4% of infection episodes were caused by Gram-positive bacteria. *Escherichia coli* and *K. pneumoniae* were the most frequently observed Gram-negative bacteria. Coagulase-negative staphylococci and Streptococcus spp. were the most prevalent Gram-positive pathogens associated with BSI in liver cirrhosis patients. In our early study, *Escherichia coli* and coagulase-negative staphylococcus were the main pathogens in SBP^23^. The results of the present study are consistent with those of previous investigations. Brandolini *et al*. reported that 41.9% of BSI cases in patients with liver disease were associated with Gram-positive bacteria^24^, and Kang *et al*. reported that *S*. *aureus* represented the main pathogen for bacteraemia^25^. Thus, from the view of antibiotic management, it is necessary to consider that Gram-positive bacteria are responsible for infection.

Nosocomial infections continue to pose a major challenge in the clinical setting. There is growing evidence indicating that nosocomial infections are associated with high drug resistance and poor prognosis^12^. This may be attributed to the diverse epidemiology, causative pathogens, and the immunocompromised nature of the patients themselves. In a similar study, Hoenigl *et al*. demonstrated that *E. coli* and *S. aureus* were the most frequently isolated pathogens, while Enterococcus spp., Candida spp., Pseudomonas spp., Enterobacter spp., and coagulase-negative staphylococci were isolated more frequently among those with hospital-acquired BSIs^26^. The results of our analysis demonstrated that the distribution of Gram-negative bacteria clearly differed between the two groups (community-acquired and nosocomial infection groups). In addition, we found that the distribution of Gram-positive bacteria did not significantly differ according to the infection source, except for that of *S*. *pneumoniae*.

Empirical antibiotic management is critical for good clinical outcomes of BSIs in liver cirrhosis patients. Generally, intravenous third generation cephalosporins are recommended as an empirical antibiotic therapy for cirrhotic patients^7^. However, in this study, we investigated the sensitivity of the isolated pathogens to commonly used antibiotics. The results revealed that Gram-negative pathogens exhibited high sensitivity to imipenem, meropenem, amikacin, cefoperazone/sulbactam, and piperacillin/tazobactam, regardless of their ESBL status. Vancomycin and teicoplanin strongly suppressed Gram-positive bacterial infections. Thus far, carbapenems represent the last line of treatments of multidrug-resistant Gram-negative pathogens in empirical treatment^27^. However, various studies have reported that carbapenems are associated with severe nephrotoxicity and ototoxicity, and excessive use of carbapenems may promote the prevalence of pathogens resistant to these drugs, resulting in serious outcomes^28, 29^. Thus, cefoperazone/sulbactam and piperacillin/tazobactam should be used for the initial empirical treatment of BSIs. However, the methicillin resistance rate was 23.8%, and *Enterococcus spp*. accounted for 9.3% of the Gram-positive bacteria in our study. The recently issued Infectious Diseases Society of America clinical practice guidelines recommend vancomycin for the treatment of bacteraemia caused by MRSA^30^. Thus, in the case of response failure, initial empirical antibiotics should be changed, and vancomycin or teicoplanin may be suitable choices.

In the current study, 30-day mortality was used to estimate the outcomes of BSI in liver cirrhosis patients. The results of this study suggested that both liver disease and infection played pivotal roles in the prognosis of the study population. Stepwise logistics regression analysis demonstrated that presentation with liver failure, liver cancer, septic shock, presence of complications, Child-Pugh grade, administration of appropriate antibiotics within 24 h, ICU admission, nosocomial infection, and Gram nature of the bacteria were independent factors correlated with clinical outcomes of BSI in liver cirrhosis patients.

Our study has several limitations. First, the study was purely observational, and clinical parameters, such as hepatic encephalopathy, depended on the judgement of physicians. Second, this study was conducted from data at a single centre. Therefore, the results obtained from this study need to be verified in a prospective multicentre study with a large sample size. Lastly, antibiotic resistance patterns might be different in other parts of the world (restricted generalizability).

In conclusion, the 30-day mortality of liver cirrhosis patients presenting with BSIs was independently correlated with liver failure, liver cancer, septic shock, presence of complications, Child-Pugh grade, administration of appropriate antibiotics within 24 h, ICU admission, nosocomial infection, and Gram nature of the bacteria. Gram-negative bacteria were the major pathogens responsible for BSIs in liver cirrhosis patients, but Gram-positive pathogens have become increasingly common. The choice of initial empirical antibiotics should be based on the type, severity and origin of the infections and on the local epidemiological data on antibiotic resistance.

## Methods

### Study population

This was a retrospective cohort study. The study protocol was approved by the Ethics Committee of our hospital, and informed consent was waived. This retrospective study included the records of patients with liver cirrhosis patients who developed BSIs in Beijing 302 Hospital from October 2010 to January 2015. The following inclusion criteria were applied for the patients screened for recruitment to this study: (1) the study population was adults over 18 years of age; (2) the patients visited the hospital for liver cirrhosis and presented with community-acquired or nosocomial (after 48 h or more since admission) BSIs; and (3) the clinical and demographic data of the patients, such as age, gender, hospitalization information, and BSI data, were available. In cases of patients who developed multiple BSIs during their hospital stay, only the first episode was used for analysis. All methods were performed in accordance with the relevant guidelines and regulations.

### Diagnosis standard

Diagnosis of liver cirrhosis was established by histological examination or by clinical, analytical, and ultrasonographic findings^3^. Non-infectious complications of cirrhosis (ascites, hepatorenal syndrome, hepatic encephalopathy) and hepatocellular carcinoma were defined in patients using criteria from the European Association for the Study of the Liver and International Ascites Club^31^. BSI was defined as the growth of a non-common skin contaminant from ≥1BCs (Blood Cultures) and of a common skin contaminant (e.g., diphtheroids, Bacillus species, Propionibacterium species, or micrococci) from ≥2BCs drawn on separate sites. To distinguish between true BSIs and contamination, each positive BC was analysed during review of the medical and microbiology records to confirm that it represented true infection. Spontaneous bacterial peritonitis (SBP) was defined as the presence of ≥250 PMN/mm3 in ascitic fluid along with/without a positive ascitic fluid culture^31^.

Patients who showed infection within 48 h of hospital admission were considered to have community-acquired infections, while those who presented with infection after 48 h of admission were considered as having nosocomial infections. The source of the BSI in each patient was determined on a clinical basis. Sources of BSI, such as lung, urinary tract, and abdomen, were defined as previously described^32, 33^. Sources of BSI were designated as culture confirmed (if the same organism was isolated from another site) or suspected (if clinical findings of infection were seen without microbiological proof). A case was regarded as a primary bacteraemia when no overt infection focus other than the bloodstream was identified.

### Blood culture and antibiotic susceptibility test

Blood samples were drawn from the subjects for antimicrobial susceptibility testing (AST). Briefly, 10 mL of blood was drawn under aseptic conditions, and the blood sample was cultured both aerobically and anaerobically with both Bact/Alert3D anaerobic and aerobic blood culture bottles (bioMerieux) at the patients’ bedside. Bacteria were inoculated into Columbia blood agar and China blue agar plates. After culture, a single colony was isolated and identified using an automated VITEK2 system (bioMerieux). The cells were tested for antimicrobial susceptibility using the Kirby-Bauer or MIC method. *Escherichia coli* (ATCC25922) and *Staphylococcus aureus* (ATCC25923) were used as the strains for quality control. Antimicrobial susceptibility testing were performed according to the recommendations of the Clinical and Laboratory Standard Institute (CLSI)^34^.

### Treatment

Empirical antimicrobial therapy was defined as the administration of antimicrobial agents after collecting the first set of positive blood cultures. In most cases, patients received a third-generation cephalosporin or piperacillin/tazobactam. In patients with a history of colonization or multidrug-resistant bacterial infection, carbapenem and vancomycin were the preferred drugs of choice. Antimicrobial therapy was considered appropriate if the drug used could inhibit the activity of the isolated pathogens in the antimicrobial sensitivity test *in vitro*. In case the drugs were not effective against the selected pathogen, a different antimicrobial agent was administered.

### Data collection

Data were collected from the medical records of the patients. The collected information included the demographic characteristics (gender and age), hospitalization unit, cause of cirrhosis, Child-Pugh score, BSI data (history of the past two years, source of BSI, days hospitalized before BSI onset, initial symptoms, complications, septic shock), bacterial distribution, drug sensitivity test results, and empirical antibiotic regimens. The 30-day mortality was counted from the first day of positive blood cultures and then used to evaluate the outcomes of BSI in liver cirrhosis patients. If the patients were discharged before 30 days, the author would call the patients at home at 30 days after infection.

### Statistical analyses

SPSS version 18.0 (SPSS, Chicago, IL, USA) was used for the statistical analyses in this study. Continuous data are presented as the means ± standard deviation and were analysed using Student’s *t* test. The chi-square test was used for categorical data analyses. Patient information was recorded in a standardized data form and compared based on the patient’s survival status within 30 days of the occurrence of infection. The stepwise logistic regression model was applied to identify the risk factors and independent risk factors for 30-day mortality. Variables in the univariate analysis (P < 0.1) and variables with clinical significance were entered into a multivariate logistic regression analysis using stepwise selection. The goodness of fit was tested with the Hosmer–Lemeshow test, which revealed that the model was of adequate fit (P = 0.813). *P* values less than 0.05 were considered statistically significant.
